# The influence of AKT isoforms on radiation sensitivity and DNA repair in colon cancer cell lines

**DOI:** 10.1007/s13277-013-1465-9

**Published:** 2013-12-14

**Authors:** Sara Häggblad Sahlberg, Ann-Sofie Gustafsson, Prathyusha N. Pendekanti, Bengt Glimelius, Bo Stenerlöw

**Affiliations:** 1 Biomedical Radiation Sciences, Uppsala University, 75185 Uppsala, Sweden; 2 Section of Oncology, Department of Radiology, Oncology and Radiation Science, Rudbeck Laboratory, Uppsala University, 75185 Uppsala, Sweden

**Keywords:** AKT1, AKT2, DNA-PKcs, MRE11, Radiation, Colorectal cancer

## Abstract

In response to ionizing radiation, several signaling cascades in the cell are activated to repair the DNA breaks, prevent apoptosis, and keep the cells proliferating. AKT is important for survival and proliferation and may also be an activating factor for DNA-PKcs and MRE11, which are essential proteins in the DNA repair process. AKT (PKB) is hyperactivated in several cancers and is associated with resistance to radiotherapy and chemotherapy. There are three AKT isoforms (AKT1, AKT2, and AKT3) with different expression patterns and functions in several cancer tumors. The role of AKT isoforms has been investigated in relation to radiation response and their effects on DNA repair proteins (DNA-PKcs and MRE11) in colon cancer cell lines. The knockout of AKT1 and/or AKT2 affected the radiation sensitivity, and a deficiency of both isoforms impaired the rejoining of radiation-induced DNA double strand breaks. Importantly, the active/phosphorylated forms of AKT and DNA-PKcs associate and exposure to ionizing radiation causes an increase in this interaction. Moreover, an increased expression of both DNA-PKcs and MRE11 was observed when AKT expression was ablated, yet only DNA-PKcs expression influenced AKT phosphorylation. Taken together, these results demonstrate a role for both AKT1 and AKT2 in radiotherapy response in colon cancer cells involving DNA repair capacity through the nonhomologous end joining pathway, thus suggesting that AKT in combination with DNA-PKcs inhibition may be used for radiotherapy sensitizing strategies in colon cancer.

**Electronic supplementary material:**

The online version of this article (doi:10.1007/s13277-013-1465-9) contains supplementary material, which is available to authorized users.

## Background

Colorectal cancer is the third most frequent cancer form in the world and also the third most common reason for cancer death. Although surgery is the primary treatment, radiotherapy and/or chemotherapy are used preoperatively or postoperatively to reduce tumor burden and to diminish recurrence risk [1]. Since not all patients will benefit from chemoradiation therapy [2], there is a great need to find new drugs with radiosensitizing properties. Understanding the molecular mechanisms of this radiosensitivity is essential for developing more effective radiotherapy treatments. Most colorectal cancers initially respond to chemotherapy, although there is a high development of drug resistance that can be linked to mutations in the DNA repair mechanism [3, 4].

AKT (also known as Protein Kinase B, PKB) is an important serine/threonine kinase in the cell signaling downstream of several growth factors, cytokines, and in response to exposure of drugs and ionizing radiation. It is involved in survival, growth, proliferation, glucose uptake, metabolism, and angiogenesis [5]. There are three isoforms of AKT (AKT1, AKT2, and AKT3) that are located on separate chromosomes and are believed to have different physiological functions, properties, and expression patterns [6, 7]. AKT isoform knockout mice have shown that suppression of AKT1 induces a reduction of body and cell size, AKT2 knockouts show diabetes mellitus-like syndrome, and AKT3 deletion causes smaller brain size and *corpus callosum* disorganization [8, 9]. Variations in AKT expression patterns, mutations, and roles of different isoforms have been observed in various cancer cell lines [10]. AKT1 may function as an oncogene and AKT3 as a tumor suppressor [11], and AKT mutations have been detected in human colorectal cancer (AKT2) and lung tumors (AKT1 and AKT3). AKT is also hyperactivated in several cancer forms and is associated with resistance to radiotherapy and chemotherapy [12].

Cells exposed to ionizing radiation acquire DNA damage such as DNA double strand breaks (DSBs), which stimulate the cells to induce signaling responses including cell cycle arrest, DNA repair, or apoptosis. The main DNA DSB repair pathways are nonhomologous end joining (NHEJ) and homologous recombination (HR) repair. The NHEJ pathway ligates the DNA ends without a long homologous DNA template. HR repair requires a homologous DNA template to be able to repair the DSBs and is therefore most active in late S/G2 phase. Both these processes are complex and require several proteins functioning at different stages in the DNA repair and radiation response [13, 14].

The catalytic subunit of nuclear DNA-dependent protein kinase (DNA-PKcs) is involved in the NHEJ pathway of DNA repair [15]. Previous studies have shown that there are important interactions between AKT and DNA-PKcs. AKT1 has been suggested to act downstream of DNA-PKcs in the DNA damage response signaling cascade, independent of ATM (ataxia telangiectasia mutated), where it provides a prosurvival signal by affecting transcriptional p21 regulation [16]. On the other hand, it has been shown that suppression ofAKT1 by siRNA reduced the phosphorylation of DNA-PKcs (Thr2609), which indicates that DNA-PKcs is instead downstream of AKT1 [17]. Furthermore, recent findings suggest that meiotic recombination 11 (MRE11), a DSB sensor protein, promotes AKT phosphorylation in response to radiation-induced DSB [18, 19]. Thus, AKT seems to interact with proteins with distinct functions in DSB recognition and repair, but knowledge of the role of individual AKT isoforms in the DNA damage response is limited.

The interactions between AKT and DNA-PKcs and MRE11 are probably dependent on a number of factors, such as celltype, genotype, and microenvironment. Previous studies have used AKT inhibitors, which are somewhat unspecific, or siRNA against AKT, which does not deplete the expression completely. In this study, two colorectal cancer cell lines, HCT116 and DLD-1, were used in which the AKT isoforms, AKT1 and AKT2, have been knocked out with no residual protein expression, which enables the analyses of the different AKT isoforms to be more reliable. The two colon cancer cell lines, HCT116 and DLD-1, have mutated *PI3KCA* and *KRAS* genes. These mutations are also common in colorectal cancer patients [20, 21]. Further, the DLD-1 cell line has a p53 mutation, and the HCT116 cell line has a MRE11 mutation. Mutations in MRE11 are common in microsatellite-unstable colorectal cancer and cause a higher sensitivity to radiation. HCT116 cells have defective MRE11 protein that lacks exons 5-7, leading to defective 3′-5′ exonuclease activity. However, it still possesses the ability to bind to DNA. These mutations are known to cause abnormal cell signaling and have to be considered when studying protein interactions and evaluating future therapies.

This study investigated how the AKT isoforms influence radiation sensitivity and affect the DSB repair rate as well as their interaction with MRE11 and DNA-PKcs. In addition, it explored how the interaction between EGFR and DNA-PKcs is affected by AKT depletion after exposure to ionizing radiation. Since the microenvironment may also play an important role in therapy response, both high and low concentrations of serum were used in the cell culture media.

## Material and methods

### Cell culture

The colon cancer cell lines DLD-1 and HCT116 X-MAN™ isogenic cell lines were obtained from Horizon Discovery Ltd. with the different AKT isoforms genetically knocked out (parental, AKT1 KO, AKT2 KO, and AKT1 and AKT2 double KO). DLD-1 parental and HCT116 parental express both AKT1 and AKT2, however not AKT3. The cells were cultured in 75 cm^2^ culture flasks (Nunclon Surface, Roskilde, Denmark) in McCoy’s 5A medium (Flow Irvine, UK) with 10 % fetal bovine serum (Sigma Aldrich), 2 mML-glutamine, 100 IU/ml penicillin, and 10 μg/ml streptomycin all from Biochrom Kg, Berlin, Germany. All cells were cultured in a humidified incubator with 5 % CO_2_ at 37 °C and trypsinized with trypsin-EDTA (0.25 % trypsin and 0.02 % EDTA, Biochrom Kg).

### Irradiation

Cells were irradiated with γ-radiation ^137^Cs source (Gammacell® 40 Exactor,BestTheratronics, Ottawa, Canada) at a dose rate of 1 Gy/min. The radiation dose was optimized for the assay performed.

### siRNA transfection

The cells were seeded in antibiotic-free cell culture media and incubated overnight at 37 °C with 5 % CO_2_. Transfection was made according to Thermo Scientific DharmaFECT siRNA transfection protocol with siRNA against DNA-PKcs (ON-Target SMART pool, PRKDC with DharmaFECT1) or against MRE11 (ON-TARGET SMART pool, MRE11A, with DharmaFECT2). The mock treatments were made with ON-TARGET plus Non-targeting Pool and the corresponding DharmaFECT solution. Three days after transfection, the cells were used for analysis.

### Western blotting for cell signaling

Cells were cultivated in 3 cm petridishes for at least three doubling times prior to exposure to radiation. Lysates were prepared in posttreatment by washing the cells with ice-cold PBS followed by addition of 10, 000, 000 cells/ml lysis buffer containing 1 % Tween-20, 20 mM Tris (pH 8.0), 137 mM NaCl, 10 % glycerol, 2 mM EDTA, 1 mM activated sodium orthovanadate (Sigma), and protease inhibitor cocktail (P8340, Sigma) and incubation on ice for 30 min. Lysates were centrifuged for 10 min in 4 °C. The supernatant was transferred to new tubes, and the pellet was discarded. The protein concentration of the lysate was determined by BCA protein assay (Pierce). Equal amounts of protein were loaded on an SDS PAGE and afterward transferred to a nitrocellulose membrane by wet blotting. The nitrocellulose membrane was blocked for 1 h in 5 % BSA, PBS and then incubated with the primary antibody overnight at 4 °C. Antibody specific for DNA-PKcs (ab1832), phospho-Ser2056-DNA-PKcs(ab18192), and phospho-Thr2609-DNA-PKcs (ab18356) were from AbCam (Cambridge, UK). Antibody against MRE11 (PC388) was from Calbiochem (EMD Millipore Corporation, Billerica, MA, USA). AKT1(sc55523 and AKT2 sc5270) were purchased from Santa Cruz Biotechnology (Santa Cruz, CA, USA), and antibodies recognizing the phosphorylated forms of AKT phospho-Ser-473/474AKT (9271) and AKT phospho-Thr308/309 (9275) were from Cell Signaling Technology (Beverly, MS, USA). Antibody against β-actin (A5441) was from Sigma-Aldrich (St. Louis, USA). After washing in PBS with 1 % Tween-20, the membrane was incubated with horseradish peroxidase-labeled secondary antibody (626520 and 656120) (Invitrogen) for 1 h at room temperature. Immunoreactive bands were visualized in a CCD camera (SuperCCD HR, Fujifilm, Japan) after treatment with electrochemiluminescent solution (Immobilon) for 5 min.

### Immunohistochemistry with PLA

Proximity ligation assay (PLA) detects proteins that are in close proximity/interacting with each other. Primary antibodies (from different species) recognize the proteins of interest and species-specific secondary antibodies, so called PLA probes, with a unique short DNA strand attached to it, bind to the primary antibodies. When the proteins are interacting, the PLA probes are in close proximity, and the DNA strands can, when hybridized with connector oligos, form circled formed DNA oligonucleotides. The circular DNA is amplified via rolling circle amplification to hundredfold replication of the DNA circle and fluorochrome-labeled complementary oligonucleotide probes highlight the product [22]. Cells were seeded on 8-well chamber slides (Nunc) in PEST-free cell culture media (McCoy’s, Sigma) and treated with siRNA or mock after 24 h. Three days after transfection, the cells were exposed to radiation (10 Gy), fixated in ice-cold ethanol 1 h postirradiation, and finally, dipped in acetone. Complexes of EGFR and DNA-PKcs or phospho-Thr2609-DNA-PKcs with phospho-S473-AKT were detected using the Duolink Proximity Ligation kit (Olink Biosciences). Cells were incubated with antibody EGFR(1005):sc-03 (SantaCruzBiotechnology) together with DNA-PKcs (1832) (AbCam) or phosphor-Thr2609-DNA-PKcs (10B1) (AbCam) together with phospho-Ser473474-AKT (Cell Signaling). Cells were incubated with complementary oligonucleotide-conjugated anti-rabbit and anti-mouse secondary antibodies followed by ligation and rolling circle amplifications in the presence of Texas Red conjugated nucleotide. The fluorescent amplicons manifest as red fluorescent dots, with each dot representing an interaction between the two specific proteins. Cells were costained with DAPI, and images were acquired using a Zeiss Axiophot fluorescence microscope. Cell profiler image software was used to measure 600–800 nuclei per experiment [23].

### mRNA quantifications with real-time qPCR

Total RNA was extracted from three biological replicates using an RNA isolation kit (Ambion). cDNA was synthesized from 0.1 μg total RNA using RevertAid H Minus First Strand cDNA Synthesis Kit with random hexamer primers (Thermo Scientific). qPCR was performed with Maxima SYBR Green/ROX qPCR Master Mix (2X) (Thermo Scientific) with qSTARqPCR primer pairs against DNA-PKcs and MRE11 and Beta-actin (OriGene) in a Step-OnePlus Real-Time PCR system (Applied Biosciences). Data were analyzed with Applied Bioscience qPCR software.

### Cell cycle analysis

Cells were fixated with 70 % ethanol, 10 % PBS and kept at −20 °C for at least 24 h. Cells were centrifuged for 10 min, 200G at 4 °C and washed twice with PBS before incubation with 5 μg Propidium Iodine(Sigma)/0.1 % NP-40(Sigma) in PBS together with 5 μg RNase (Sigma) for 30 min at room temperature. Analysis was made with flow cytometry (BD LSRII Biosciences).

### Clonogenic assay

To study the effect on cell survival of radiation, clonogenic survival assays were performed using standard technique. The cells were preplated before radiation since this allows the cells to be undisturbed after the radiation exposure. Cells were harvested by using trypsin for cell detachment followed by counting in a Z2 Coulter Counter Analyzer (Beckman Coulter, FL, USA), and a certain number of cells (300 up to 20, 000 depending on treatment) were preplated in 25 cm^2^ tissue culture flasks with 10 ml complete medium. The cells were allowed to attach during culture conditions in humidified air with 5 % CO2 overnight to give them time to regain their cell-surface receptors after trypsinization. The following day, the cells were exposed to radiation (4 Gy). Control cultures were left unexposed and some cultures were exposed to radiation only. After 8–14 days incubation (depending on the doubling time of the cell lines), cells were washed in 1 × PBS, and fixed with 99.5 % ethanol and stained with Mayer’s Haematoxylin. Colonies containing more than 50 cells were counted manually.

The plating efficiency (PE), number of colonies formed/number of cells seeded, in the untreated control and the survival fraction (SF), number of colonies formed after treatment/number of seeded cells × PE, were calculated. All experiments were repeated in triplicate at least three times. The survival curve was analyzed using the linear–quadratic formula (SDose/S0) = exp(αD + βD2).

Detection of DNA double strand breaks by pulsed-field gel electrophoresis.

Pulsed-field gel electrophoresis (PFGE) is a method to analyze the rapid rejoining of radiation induced DNA double strand break [24]. This method was chosen over γH2AX foci formation assay since DLD-1 and HCT116 forms stacked cell clusters which make the detection of foci difficult. Cells for PFGE were plated in 3-cm dishes and labeled with 2 kBq/ml [methyl-^14^C] thymidine (Perkin Elmer) for approximately two doubling times. The dishes were put on ice 20–30 min before irradiation and were kept on ice during the entire irradiation. Cells were prepared for PFGE as described previously [25]. After irradiation and repair in incubation at 37 °C, cells were trypsinized and mixed with low gelling-point agarose (InCert, Cambrex) to a final concentration of 1.5–2.5 × 10^6^ cells/ml in 0.6 % agarose. The mixture was transferred into plug-molds. The plugs with cells were then transferred to ESP lysis buffer at 4 °C [2 % N-lauroylsarcosine (Sigma), 1 mg/ml proteinase K (Roche), all diluted in 0.5 M EDTA (Na_3_) at pH 8.0]. After >20 h, the ESP buffer was removed and replaced with 20 plug volumes HS-buffer and incubated overnight at 4 °C (HS, high salt; 1.85 M NaCl, 0.15 M KCl, 5 mM MgCl_2_, 2 mM EDTA, 4 mM Tris, 0.5 % Triton X-100, pH 7.5, Triton X-100 was added just before use). Plugs were washed in 0.1 M EDTA and once in 0.5xTBE at 4 °C prior to electrophoresis. The plugs were then loaded into wells in a chilled (4 °C) agarose gel (0.8 % SeaKem Gold, Lonza). The gel was placed into a PFGE unit (Gene Navigator, Amersham Pharmacia Biotech, Uppsala, Sweden) with 120° between the fields. Following electrophoresis, the gels were sliced at the position of the 5.7 Mbp chromosome from *S. pombe* (BMA), and ^14^C in the gel segments was measured by liquid scintillation. The fraction of radioactivity corresponding to DNA of size less than 5.7 Mbp was divided by the total radioactivity in the lane, giving the fraction of DNA <5.7 Mbp, which is a relative measure of DNA double-strand breaks.

### Statistical analysis

The data were processed with Microsoft Office Excel 2007 (Microsoft, Redmond), and all graphs were plotted in GraphPad Prism 5 (GraphPad Software, San Diego).

Statistical analysis was performed using GraphPad Prism or Excel to a perform 2-sided Student’s *t* test. A significance level of 95 % was used. This analysis evaluated whether the effects of treatments or genetic knockout were significantly different from the untreated controls.

## Results

### The influence of AKT isoforms on the expression of DNA-PKcs and MRE11

**Fig. 1:**
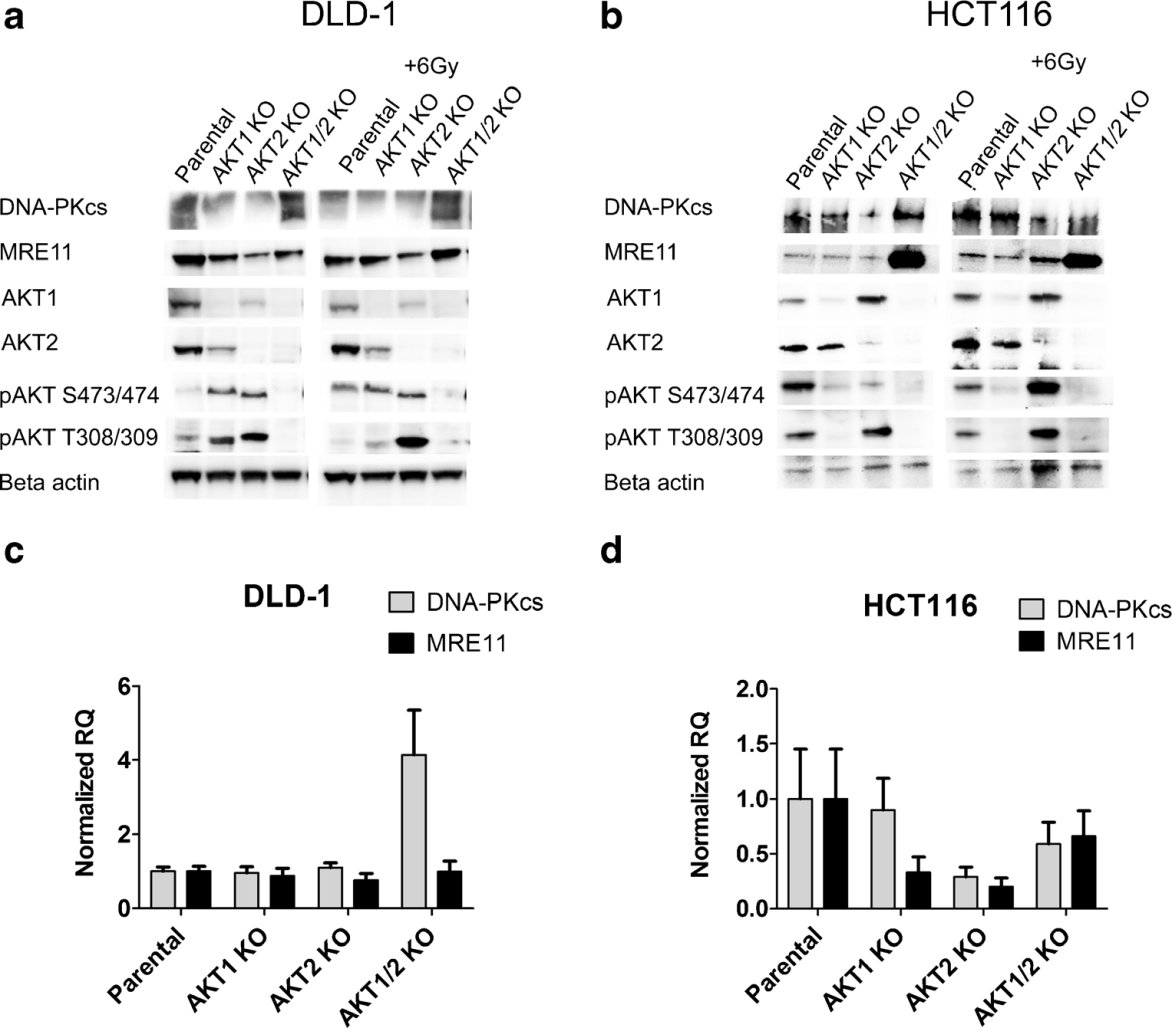
Protein expression and mRNA levels of DNA-PKcs and MRE11 are influenced by the AKT isoforms. Western blots were performed to study the protein expression and phosphorylation of the AKT isoforms, DNA-PKcs and MRE11 in the colorectal cancer cell lines DLD-1 (**a**) and HCT116 (**b**) and their corresponding AKT isogenic knockout. Cell-lysates were made before and after irradiation (1 h post IR 6 Gy). The mRNA level of DNA-PKcs and MRE11 were analyzed with qPCR in DLD-1 cells (**c**) and HCT116 cells (**d**) and their corresponding AKT isogenic knockouts. The data is from at least two biological replicates with each sample measured in triplicates using Beta-actin as reference in the delta-delta Ct model. The *error bars* represent the normalized RQ min and max

Two colorectal cancer cell lines, DLD-1 and HCT116, and their corresponding isogenic AKT isoforms knockout cell lines were used to show whether AKT1 or AKT2 were activated after exposure to ionizing radiation and their effect on the expression of MRE11 and DNA-PKcs. The DLD-1 parental cell line had an increased expression of phospho-AKT at Ser473/474 after exposure to ionizing radiation, 1 h post IR (Fig. 1a). The AKT1 and AKT2 knockout cell lines, which had a higher constitutive activation of the remaining AKT isoform compared to parental cells, had no further increase in phosphorylation after exposure to radiation. In the case of HCT116, the AKT1 KO cell line had a lower phosphorylation of AKT compared to the parental and AKT2 KO, suggesting that AKT1 is the isoform that is mainly activated in HCT116 (Fig. 1b).

The expression of DNA-PKcs and MRE11 proteins were influenced by the AKT isoforms. Single depletion of AKT1 or AKT2 resulted in lower protein levels of DNA-PKcs and MRE11 in DLD-1. However, double depletion of AKT1 and AKT2 caused an increase in the expression of DNA-PKcs and MRE11. In contrast, in the HCT116 cell-line, DNA-PKcs expression was only reduced in the AKT2 KO cells and the MRE11 expression was low in parental as well as the single AKT isoform knockout cell lines.

To gain further insight in the expression of DNA-PKcs and MRE11, mRNA levels were quantified with qPCR using the delta-delta Ct-calculation. There was a 4-fold increase in mRNA levels of DNA-PKcs in the AKT1/2 KO cell line compared to the parental DLD-1, which confirms the western blot data, but there was no difference in the mRNA level of MRE11 (Fig. 1c). In HCT116, there was a slight decrease in mRNA levels of DNA-PKcs and MRE11 in the AKT2 KO but not in AKT1 or AKT1/2 KO cell lines, which is in agreement with the western blot data (Fig. 1d). Notably, the dramatic increase in MRE11 protein in the AKT 1/2 KO cell line (Fig. 1b) seems to be completely due to posttranslational regulation such as increased protein stability or low degradation.

There were no variations in the cell-cycle distributions among the AKT isoform knockouts suggesting that the differences seen in mRNA level or protein expression/activation were not due to differences in cell-cycle (Table 1).

**Table 1:** Cell cycle distribution in cells with knockout of different AKT isoform

Cell line	G0/1	S	G2/M
DLD-1 Parental	52	28	18
DLD-1 AKT1 KO	58	23	17
DLD-1 AKT2 KO	50	26	22
DLD-1 AKT1/2 KO	55	21	23
HCT116 Parental	76	7	11
HCT116 AKT1 KO	78	6	10
HCT116 AKT2 KO	74	7	13
HCT116 AKT1/2 KO	73	5	16

### Phosphorylation of AKT is influenced by DNA-PKcs but not MRE11

**Fig. 2:**
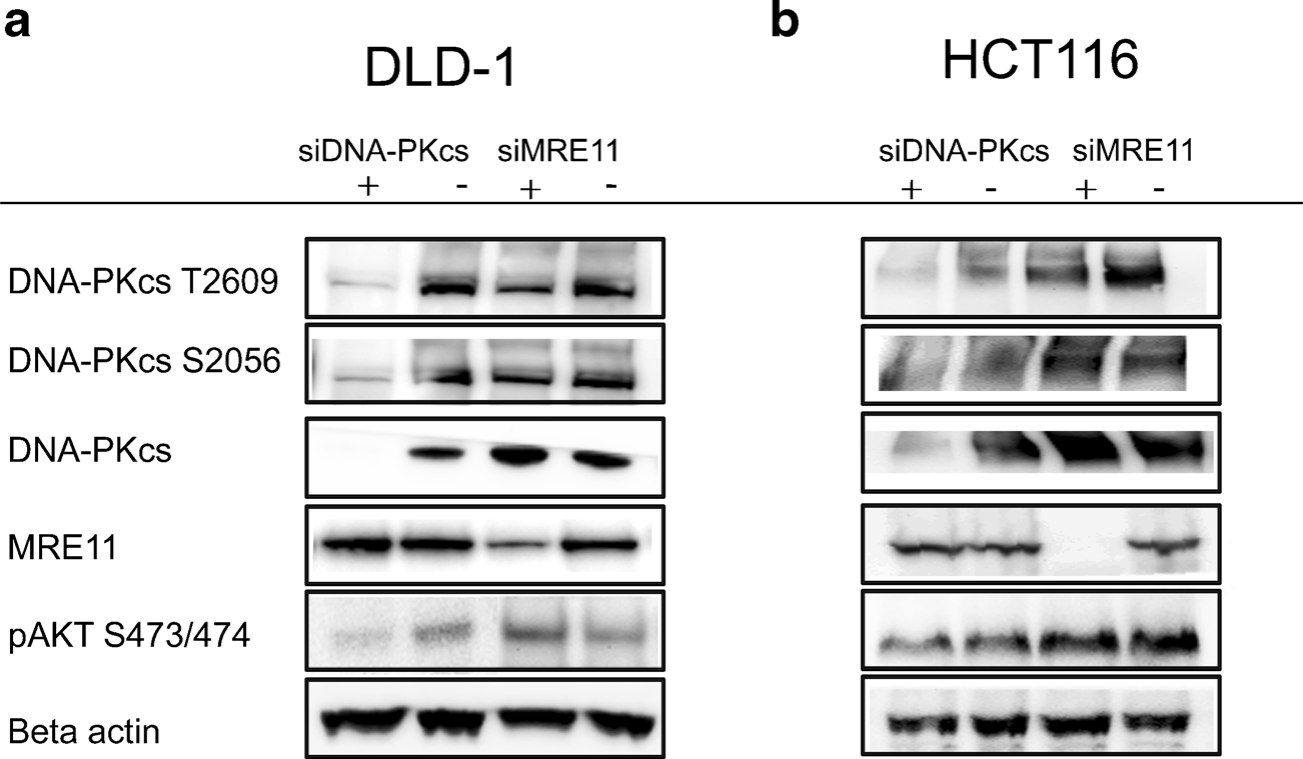
The phosphorylation of AKT is influenced by DNA-PKcs but not MRE11. DNA-PKcs and MRE11 expression was suppressed with siRNA against either DNA-PKcs or MRE11 in DLD-1 cells (**a**) and HCT116 cells (**b**) and lysates were made 1 h after irradiation (2 Gy) 3 days after transfection. The expression of AKT and DNA-PKcs and MRE11 was studied with western blot. The experiment was repeated at least twice and a representative western blot is shown

To study the interaction between AKT and the DNA repair proteins (DNA-PKcs and MRE11) further, cells were treated with siRNA against either DNA-PKcs or MRE11. In DLD-1 but not in HCT116, AKT activation was influenced by DNA-PKcs shown by a small reduction in the phospho-Ser473/474-AKT after treatment with siRNA against DNA-PKcs. DNA-PKcs might therefore be one of the PDK2 proteins which activate AKT (Fig. **2**a and b). Treatment with siRNA against MRE11 had no effect on the phosphorylation of AKT and is therefore not considered as PDK2 in these cell lines. However, suppression of MRE11 caused a reduction in the expression of phosphorylated Thr2609-DNA-PKcs, suggesting that MRE11 and DNA-PKcs interact with each other.

### Phospho-Thr2609-DNA-PKcs interacts with phospho-Ser473-AKT1 and phospho-Ser474-AKT2

**Fig. 3:**
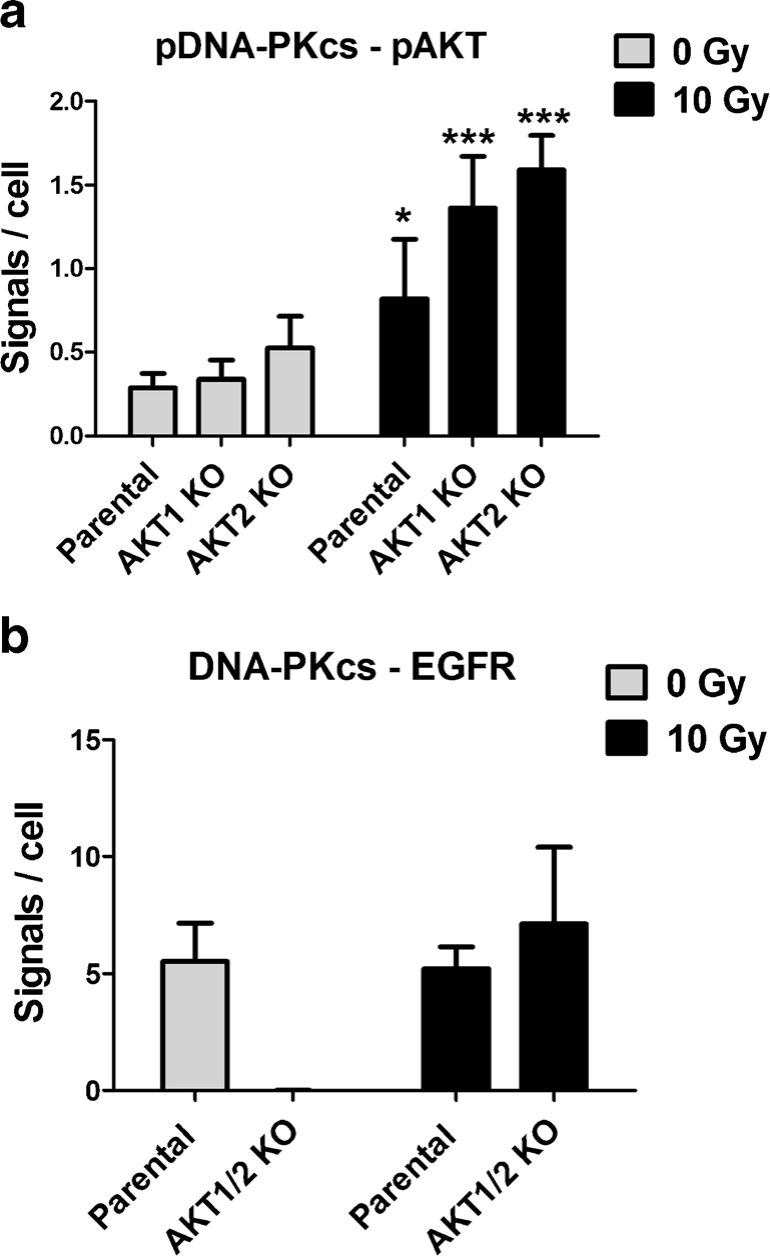
DNA-PKcs is in close proximity to phospho-Ser473/474-AKT or EGFR in DLD-1 cells. DLD-1 and its corresponding AKT isotype knockout cells were exposed to radiation (1 h post IR 10 Gy) and analyzed with the proximity ligation assay (PLA) of the particular proteins. The interaction between phospho-Thr2609-DNA-PKcs with phospho-Ser473/474-AKT A) as well as between DNA-PKcs and EGFR B) were analyzed before and after irradiation (1 h, post IR10 Gy). The *error bars* represents the standard deviation from five measurements. The difference was analyzed with Student’s *t* test where *P < 0.05, **P < 0.01, and ***P < 0.001

As indicated above, AKT affects the expression of DNA-PKcs, which in turn has a role in the activation of AKT. Proximity Ligation Assay (PLA) was performed to define better how DNA-PKcs and AKT interacts and how this relates to the presence of AKT. PLA detects proteins in close proximity and when the specific proteins are close enough, there will be a fluorescent spot which can be seen with microscopy. There was a low but detectable number of phospho-Thr2609-DNA-PKcs and phospho-Ser473/474-AKT in close proximity, shown as spots, in the DLD-1 parental, AKT1, and AKT2 KO cell lines, indicating that DNA-PKcs interacts with both AKT1 and AKT2. This interaction was most prominently detected in the cell nucleus. In unirradiated cells there was no difference between the parental and the AKT1 KO or AKT2 KO cell lines. After exposure to radiation (1 h post-IR) this interaction increased significantly, with slightly higher number of spots in the AKT1 and AKT2 KO cell lines compared to the parental cell line (Fig. 3a).

### EGFR interaction with DNA-PKcs is dependent on AKT

EGFR may directly or indirectly interact with DNA-PKcs; and therefore, the amount of EGFR in close proximity to DNA-PKcs was analyzed. The majority of the spots were detectable in the cell nuclei. There was no difference in the number of EGFR-DNA-PKcs spots in the parental cell line before and after radiation (Fig. 3b). In contrast, DLD-1 AKT1/2 KO cells have no DNA-PKcs-EGFR in close proximity to each other under normal conditions. However, 1 h after exposure to radiation the number of DNA-PKcs and EGFR in close proximity were the same in the AKT1/2 KO as in the parental cell line, suggesting that this interaction was triggered by radiation even in the absence of AKT.

### Both AKT1 and AKT2 contribute to survival following radiation exposure

**Fig. 4:**
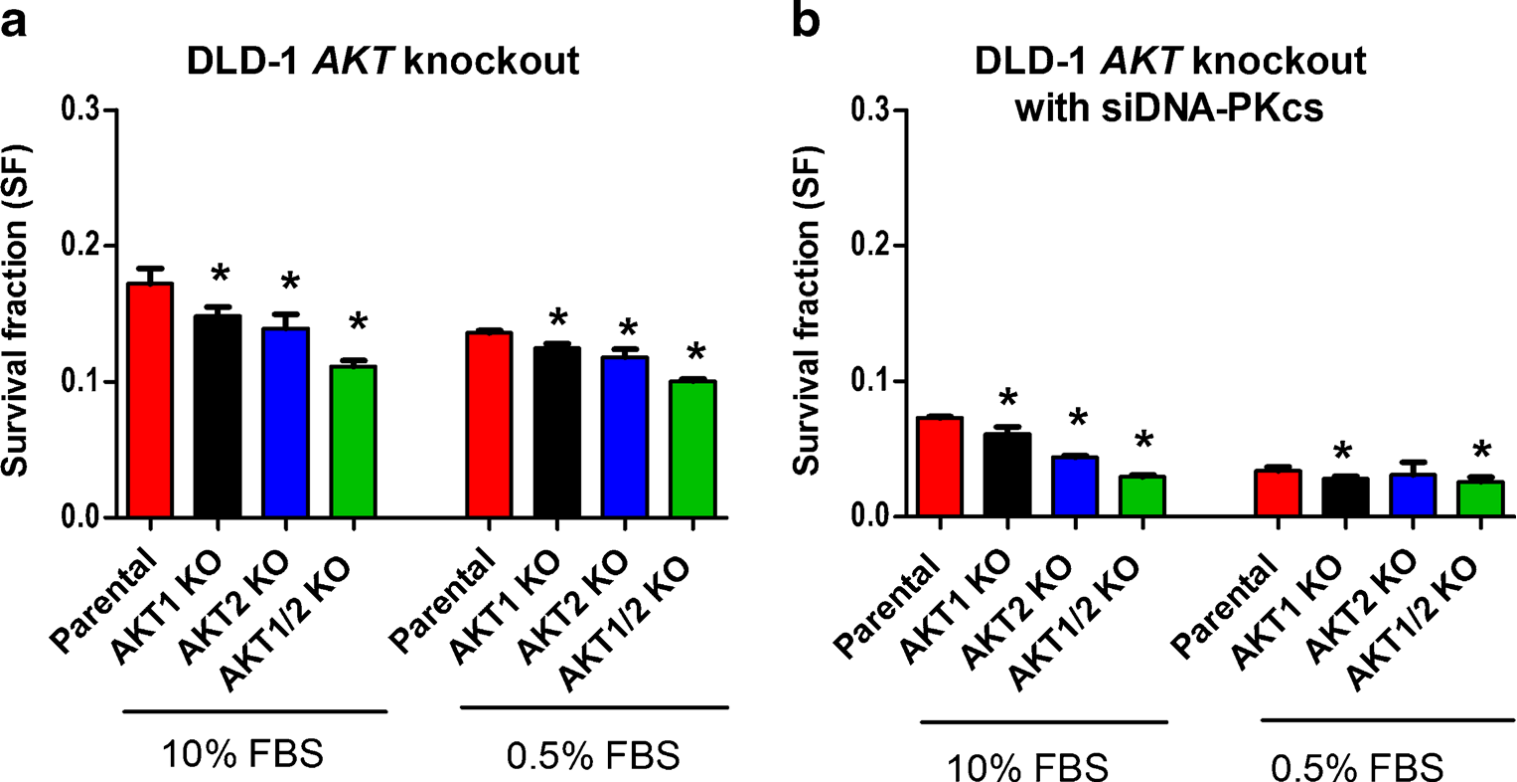
Radiation survival is dependent on AKT, DNA-PKsc and serum concentration. Radiation sensitivity was analyzed with clonogenic assay (4 Gy) in DLD-1 AKT isoform knockout cell lines treated with mock (**a**) or siRNA against DNA-PKcs (**b**) in 10 % FBS and 0.5 % FBS. The cells treated with 0.5 % FBS were first seeded in flasks with 10 % FBS and incubated for 24 h to allow attachment before changing to 0.5 % FBS. The cells were incubated in 24 h in 0.5 % FBS before radiation and kept in the same media after treatment. The *error bars* represents the standard deviation from at least three experiments. Student’s *t* test evaluated if there were any significant differences in SF between the parental and the AKT KO cell lines with *P < 0.05, **P < 0.01, and ***P < 0.001

To evaluate if the radiation sensitivity was dependent on the different AKT isoforms, cells were exposed to 4 Gy and analyzed with clonogenic assay. There was a significant (*p* < 0.05, Student’s *t* test) increase in the sensitivity to radiation in the DLD-1 single AKT1 and AKT2 KO and an even further increase in the double AKT1/2 KO cell line. Since growth factors are involved in the radiation response and cell survival, the radiation response was also studied in cells that were starved 24 h before radiation in 0.5 % FBS culture media. The reduction in FBS significantly (*p* < 0.05, Student’s*t* test) increased the radiation sensitivity in parental as well as single and double AKT isoform KO cells (Fig. 4a).

### Suppression of DNA-PKcs leads to higher radiosensitivity

The radiation sensitivity in DNA-PKcs suppressed cells was analyzed. Since there was a small reduction in the phosphorylation of AKT Ser473 in DLD-1 cells treated with siRNA against DNA-PKcs, an increase in radiation sensitivity was expected. The DLD-1 parental and the different AKT isoform KO cell lines were treated with siRNA against DNA-PKcs and irradiated (4 Gy) in the presence of 10 % FBS or 0.5 % FBS. Suppression of DNA-PKcs caused a reduction (*p* < 0.05, Student’s *t* test) in the survival fraction, and in combination with starvation (0.5 % FBS) the radiation sensitivity was further increased. The highest radiosensitivity was observed in the AKT KO cell line treated with siDNA-PKcs in 0.5 % FBS (Fig. 4b).

### The AKT isoforms influence the DSB rejoining

**Fig. 5:**
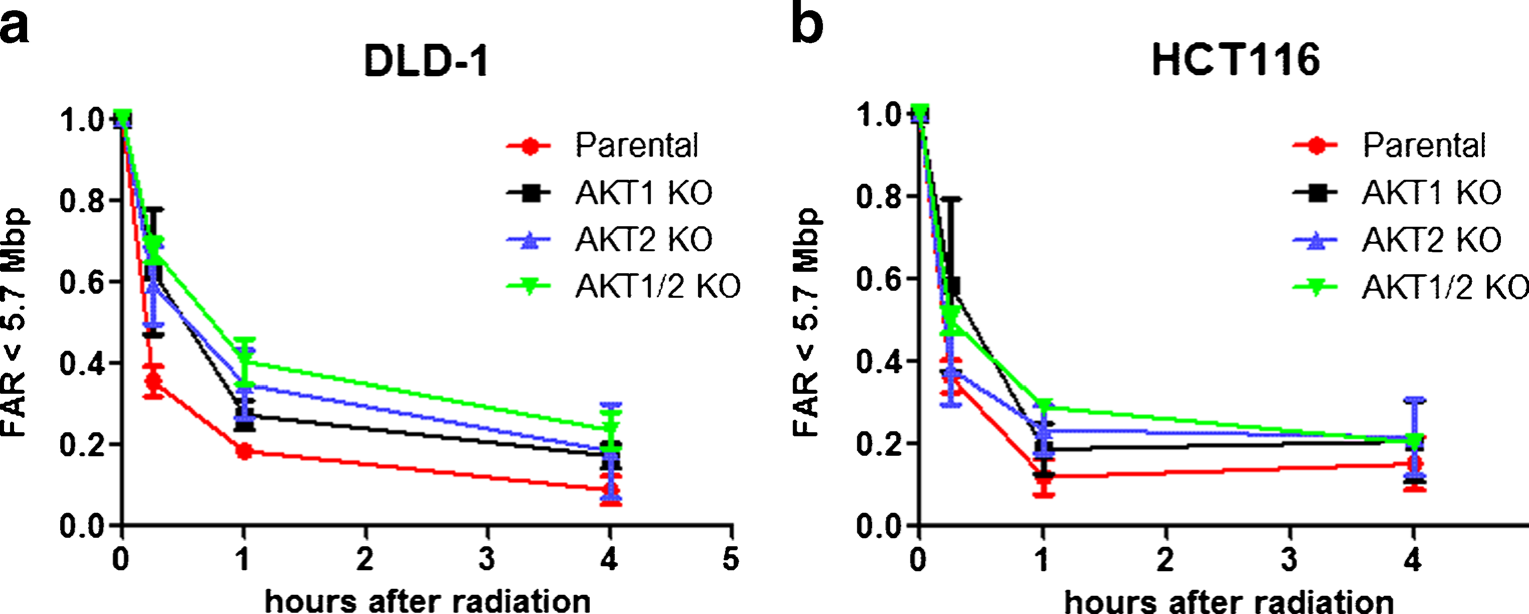
DNA double strand break-rejoining rate correlates with radiation sensitivity in DLD-1 AKT isoform deficient cell lines. DNA double strand break rejoining rate, evaluated with pulsed-field gel electrophoresis after irradiation (40 Gy), in the parental and AKT isoform knockout cell lines of DLD-1 (**a**) and HCT116 (**b**) in 10 % FBS. The *error bars* represent the standard deviation of at least two measurements

To study if radiation sensitivity correlates with an impaired DSB rejoining rate, pulsed-field gel electrophoresis was performed on the different AKT KO cell lines. In both DLD-1 and HCT116 cells, all the AKT isoform KO cell lines showed a tendency for a slower DNA repair rate compared to the parental cell line in 10 % FBS (Fig. 5a and b). The DLD-1 AKT1/2 KO cells had a significant slower DSB rejoining rate compared to the parental cells (*p* < 0.05 in Student’s *t* test).

**Fig. 6:**
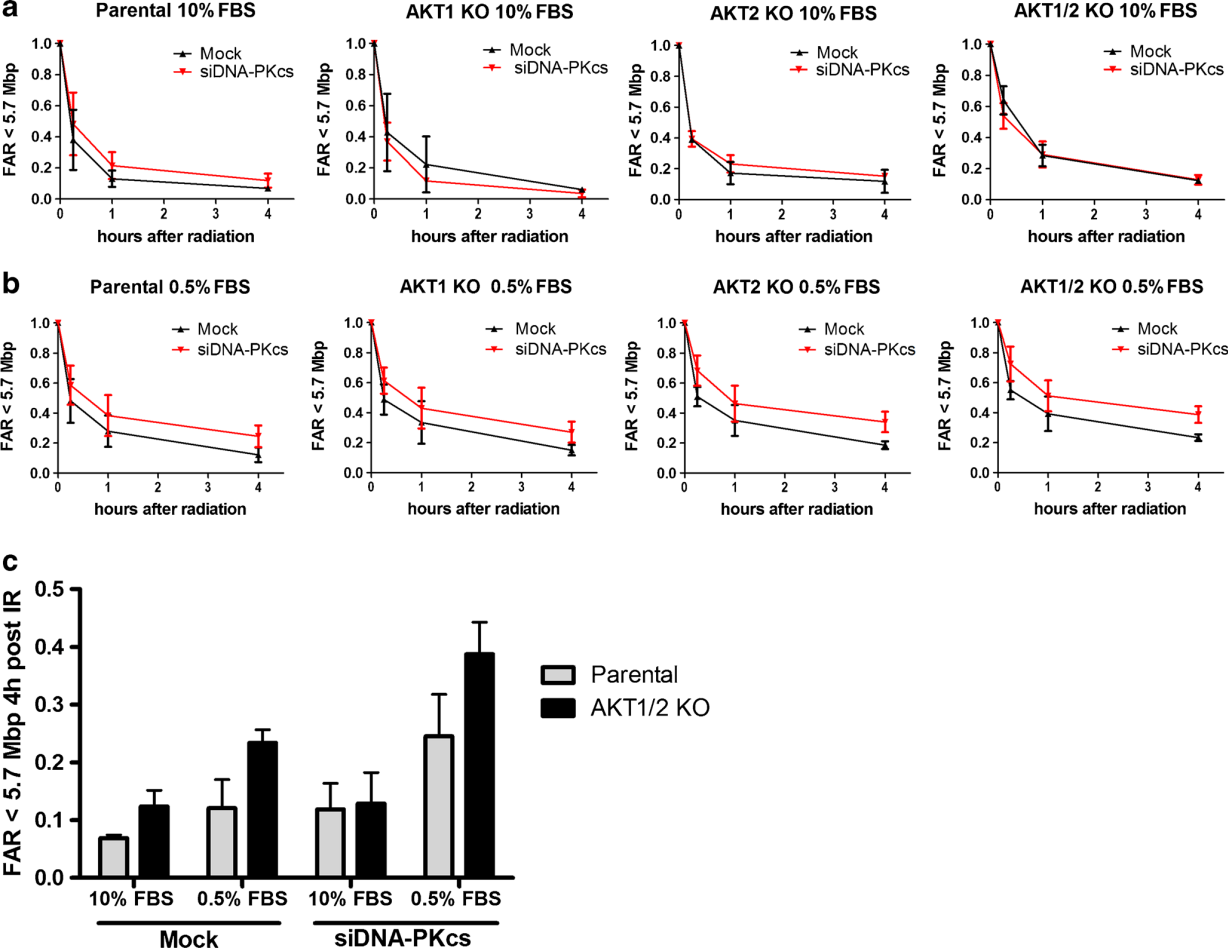
DNA double strand break-rejoining rate in AKT isoform deficient DLD-1 with suppressed DNA-PKcs expression. DNA double strand break rejoining rate, evaluated with pulsed field gel electrophoresis after irradiation (40 Gy), **a** in DLD-1 and the AKT isoform knockout cell lines at 10 % FBS and 0.5 % FBS and **b** mtreated with siRNA against DNA-PKcs. **c** Graph representing unrejoined DNA double strand breaks 4 h post irradiation in DLD-1 parental and AKT1/2 KO cell lines treated with mock or siDNAPK-cs in 10 % or 0.5 % FBS. The *error bars* represent the standard deviation of at least two measurements. Student’s t-test evaluated if there were any significant differences in DNA rejoining between the parental and the AKT KO cell lines with *P < 0.05, **P < 0.01, and ***P < 0.001

The DSB rejoining in siDNA-PKcs-treated cells was impaired in serum-starved cells (0.5 % FBS). However, this effect was not significant in 10 % FBS (Fig. 6a and b). AKT1/2 KO in combination with siDNA-PKcs in 0.5 % FBS significantly (*p* < 0.05) reduced the DNA rejoining rate compared to parental in 0.5 % FBS. On the other hand, this was not seen in 10 % FBS.

Even though MRE11 affects the activation of DNA-PKcs, a suppression of MRE11 did not reduce the DNA rejoining rate in any of the cell lines in either 10 or 0.5 % FBS. In HCT116, neither suppression of DNA-PKcs nor MRE11 reduced the DSB rejoining significantly at either 10 or 0.5 % FBS (data not shown).

## Discussion

AKT is mainly activated via the Phosphotidyl Inositol-3 Kinase (PI3K) pathway where PI3K activates PIP2 to PIP3, which in turn will bind to the PH-domain of AKT and alter the conformation of AKT to allow subsequent phosphorylation at a threonine site (Thr308 for AKT1 and Thre 309 for AKT2) and a serine site (Ser473 for AKT1 and Ser474 for AKT2). Phosphoinositide Dependent Kinase-1 (PDK1) is a known serine/threonine kinase which phosphorylates AKT at Thr308. However, the mechanism of phosphorylation at Ser473 is not clear and several theories have been proposed. The Ser473 is suggested to be either phosphorylated by PDK1 or by some unknown protein named PDK2. Among possible PDK2 proteins are ILK-1, mTOR, ATM, DNA-PKcs, and MRE11 [26]. AKT may also be activated through a PI3K-independent manner via many different pathways including other phosphorylation sites such as tyrosines. There are also indications that Ser473 is autophosphorylated. It has previously been shown by us and others that AKT activation is radiation dose and time dependent [27], and it is believed to be involved in the DNA repair after exposure to ionizing radiation [16, 28].

Previous studies have tried to evaluate the importance of the different AKT isoforms in terms of radiation response using siRNA treatment. However, the suppression of protein expression with siRNA treatment is not complete, leaving at least 10–20 % of residual protein levels. In a study by Kim et al. on three different types of tumor cell lines, including the colon cancer cell line SW480, treatment with siRNA against AKT1 before irradiation reduced cell survival more than siRNA treatment against AKT2 or AKT3 [29]. Bozulicet al. also showed that in mouse embryonic fibroblasts (MEF) cells, treatment with siRNA against AKT1, but not AKT2 or AKT3, there was an increase in the radiation-induced apoptosis. They further noted that only AKT1 was interacting with DNA-PKcs [16].

In the present study, isogenic knockouts of AKT were used instead of siRNA, and it was shown that both AKT1 and AKT2 are involved in the response to radiation and that abolishment of either AKT1 or AKT2 isoforms alone or together increases the radiation sensitivity. It was further shown that deleting both AKT1 and AKT2 isotypes simultaneously impairs the DNA-rejoining of DSBs. Suppression of DNA-PKcs had a radiosensitizing effect, which was further increased in combination with disruption of AKT, and the PLA assay confirmed that DNA-PKcs interacts with both AKT1 and AKT2. Interestingly, suppression of DNA-PKcs with siRNA also reduced the activation of AKT in DLD-1 but not in HCT116. The two cell lines harbor different mutations that evidently affect the interaction between AKT and DNA-PKcs which should be evaluated further in future studies. The study also demonstrated the importance of serum level, which is often abrogated in tumors due to their abnormal growth.

The present study has also shown that the expressions of DNA-PKcs and MRE11 were increased when both AKT1 and AKT2 isoforms were knocked out, as seen in both DLD1 and HCT116. However, in the single AKT1 or AKT2 KO cell lines, the expression of DNA-PKcs and MRE11 was reduced, which suggests a feedback-loop causing an increase in DNA-PKcs and MRE11 expression only when both isoforms are disrupted. However, despite the increased expression of the DNA-repair proteins in the AKT1/2 KO cells, these cells still had a higher sensitivity to radiation. It has been proposed that DNA-PKcs is involved in apoptosis, and an activation of DNA-PKcs has been detected in several types of cells in the early stages of apoptosis [30]. When both AKT isotypes are deleted, there is an increase in apoptosis [31], which could explain the increase in DNA-PKcs. Interestingly, under stress conditions, such as low serum, the rejoining was impaired in DNA-PKcs-suppressed cells but not in 10 % FBS. This implies that even a low expression (<20 %) of DNA-PKcs is enough for the cell to rejoin DSB under normal growth conditions. However, the decreased cell survival indicates that DNA-PKcs have other important roles besides DSB repair, and recent data suggest that inactivation of DNA-PKcs cause multipolar spindle and mitotic catastrophe after DNA damage [32]. The findings of the present study also confirm a recent study by Reynolds et al., which showed that DNA-PKcs is not involved in rapid repair of DSBs but is instead recruited to the slow repairing DSBs, which also require Ku80 in the NHEJ process [33]. Therefore, the effect of suppressing DNA-PKcs is only seen in the clonogenic assay, which reveals the long-term effects of radiation exposure.

It is proposed that the effects in low serum level media could be due to the reduced growth factors. Recent studies have shown the importance of growth factor receptors such as EGFR and HER-2 for the function of DNA-PKcs. Activated EGFR activates AKT in the nucleus or directly activates DNA-PKcs in response to ionizing radiation [34–36]. Interestingly, the interaction between EGFR and DNA-PKcs was dependent on AKT. The PLA assay could not detect any EGFR-DNA-PKcs in close proximity in AKT1/2 KO cells. However, when the cells were exposed to radiation, the number of DNA-PKcs-EGFR spots was the same as in the parental cells, suggesting that there are other pathways and feedback loops involved to enable the cell to overcome exposure to radiation.

The DNA-PKcs activation was also influenced by MRE11, which is primarily involved in homologous recombination (HR), but it is also part of the NHEJ pathway. However, treatment with siRNA against MRE11 did not affect the DSB rejoining in either DLD-1 or HCT116.

## Conclusion

Taken together, our results present strong support for the role of both AKT1 and AKT2 isoforms in the response to ionizing radiation and their interaction with DNA-PKcs and MRE11 in colon cancer cells. Targeting all AKT isoforms in combination with a DNA-PKcs inhibitor could have therapeutic implications when used in combination with radiotherapy in colorectal cancer patients.

## Electronic supplementary material

Below is the link to the electronic supplementary material.

Supplementary S1 The radiation sensitivity and DSB rejoining in DLD-1 and HCT116 colon cancer cell lines. Radiation sensitivity of HCT116 and DLD-1 were evaluated with clonogenic assay at 0 to 6 Gy A). The survival fraction (SF) is the number of colonies divided by the number of cells seeded and normalized to the plating efficiency in the unirradiated controls. HCT116 have a survival fraction (SF) of 0.42 at 2 Gy and DLD-1 have a SF of 0.67 at 2 Gy The DNA-double strand break rejoining rate in DLD-1 and HCT116 in 10 % FBS was studied with pulsed-field gel electrophoresis at different repair times after exposure to radiation (40 Gy) B). DNA fragments smaller than 5.7 Mbp are considered unrejoined. The relative measure of DNA double-strand breaks is calculated by dividing the fraction unrepaired DNA corresponding to DNA < 5.7 Mbp, with the total DNA content for each sample. The error bars represent the standard deviation from at least three experiments. (JPEG 12 kb)

High resolution image (TIFF 2300 kb)
