# Supplementary figures and images for: The influence of AKT isoforms on radiation sensitivity and DNA repair in colon cancer cell lines
Source: Tumour Biol. 2013 Dec 14;35(4):3525–34. doi: 10.1007/s13277-013-1465-9 (PMC3980041; doi:10.1007/s13277-013-1465-9)

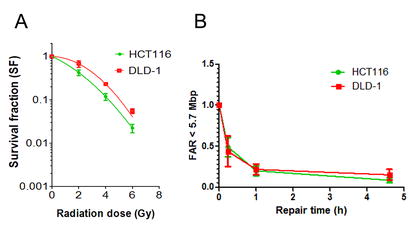

Supplement: Supplementary file 1 — The radiation sensitivity and DSB rejoining in DLD-1 and HCT116 colon cancer cell lines. Radiation sensitivity of HCT116 and DLD-1 were evaluated with clonogenic assay at 0 to 6 Gy A). The survival fraction (SF) is the number of colonies divided by the number of cells seeded and normalized to the plating efficiency in the unirradiated controls. HCT116 have a survival fraction (SF) of 0.42 at 2 Gy and DLD-1 have a SF of 0.67 at 2 Gy The DNA-double strand break rejoining rate in DLD-1 and HCT116 in 10 % FBS was studied with pulsed-field gel electrophoresis at different repair times after exposure to radiation (40 Gy) B). DNA fragments smaller than 5.7 Mbp are considered unrejoined. The relative measure of DNA double-strand breaks is calculated by dividing the fraction unrepaired DNA corresponding to DNA < 5.7 Mbp, with the total DNA content for each sample. The error bars represent the standard deviation from at least three experiments. (JPEG 12 kb) [file 13277_2013_1465_Fig7_ESM.jpg]

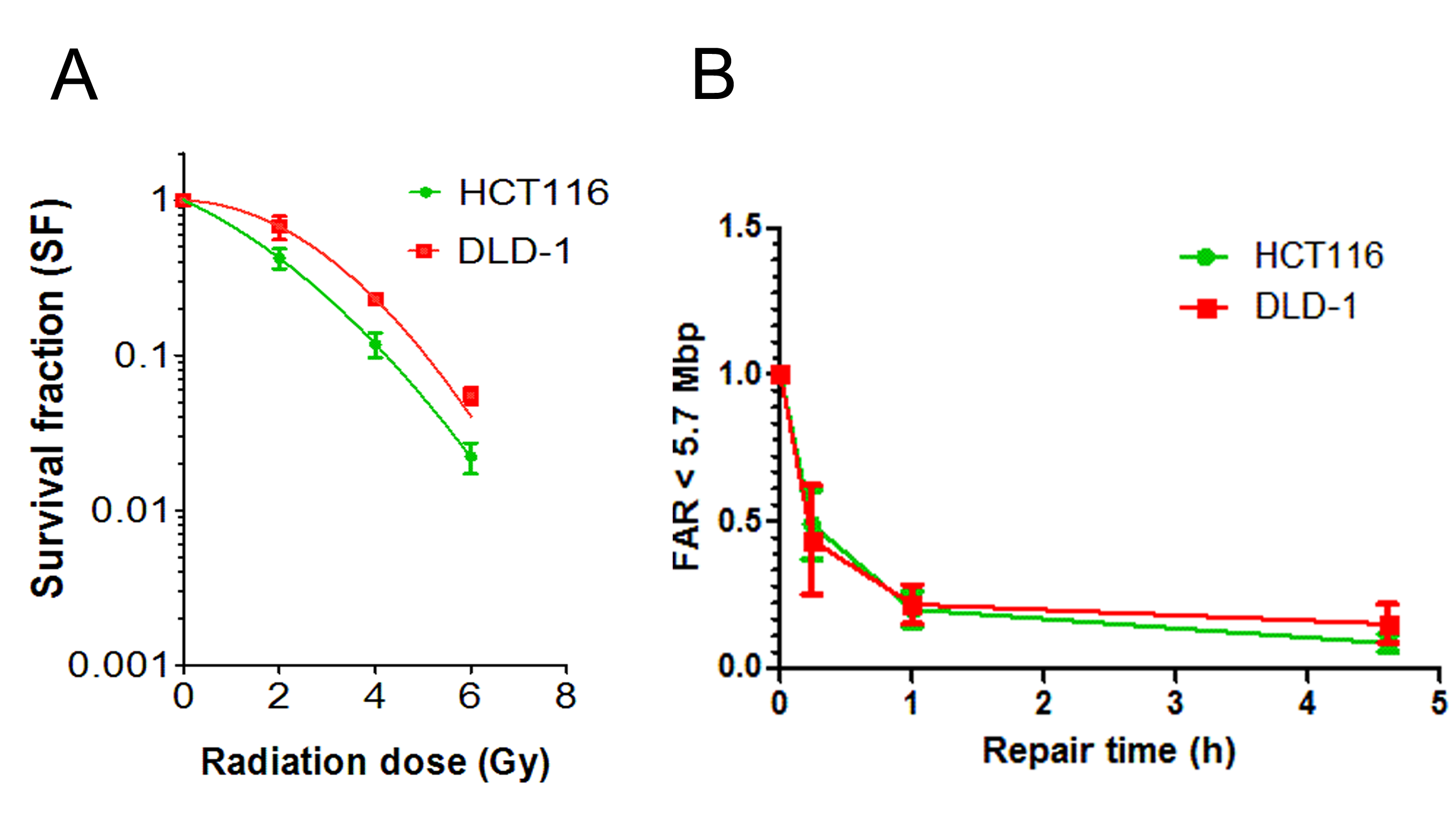

Supplement: Supplementary file 2 — High resolution image (TIFF 2300 kb) [file 13277_2013_1465_MOESM1_ESM.tif]
